# Nosewitness Identification: Effects of Negative Emotion

**DOI:** 10.1371/journal.pone.0116706

**Published:** 2015-01-22

**Authors:** Laura Alho, Sandra C. Soares, Jacqueline Ferreira, Marta Rocha, Carlos F. Silva, Mats J. Olsson

**Affiliations:** 1 Department of Education, University of Aveiro, Aveiro, Portugal; 2 Institute for Biomedical Imaging and Life Sciences (IBILI), Faculty of Medicine, University of Coimbra, Coimbra, Portugal; 3 Center for Health Technology and Services Research (CINTESIS), Faculty of Medicine, University of Porto, Porto, Portugal; 4 Department of Clinical Neuroscience, Division for Psychology, Karolinska Institutet, Stockholm, Sweden; University of Graz, AUSTRIA

## Abstract

Every individual has a unique body odor (BO), similar to a fingerprint. In forensic research, identification of culprit BOs has been performed by trained dogs, but not by humans. We introduce the concept of *nosewitness identification* and present the first experimental results on BO memory in witness situations involving violent crimes. Two experiments indicated that BO associated with male characters in authentic videos could later be identified in BO lineup tests well above chance. Moreover, culprit BO in emotional crime videos could be identified considerably better than the BO of a male person in neutral videos. This indicates that nosewitness identification benefits from emotional encoding. Altogether, the study testifies to the virtue of body odor as a cue to identify individuals observed under negative emotion.

## Introduction

No two individuals smell alike. Determinants of body odor are diet [1], age [2], hormonal status [3], parasite load [4], and foremost and pertinent to this study, genetic factors. The major histocompatibility complex (MHC) is a gene cluster that constitutes the main factor in determining immunological individuality. It also contributes to an “odor print” that is unique to each individual [5, 6]. Accordingly, humans have the ability to match body odors of monozygotic twins, even when they are living apart (i.e., exposed to different environments), which reinforces the role of odors in communicating individuality and genetic information [7]. Moreover, trained dogs can distinguish between any two individuals (possibly including monozygotic twins [8]) and have been used in forensic settings to match a scent left at the crime scene by a possible suspect [9–11].

In forensic psychology, it is common to ask witnesses to identify the culprit of a crime in a lineup of foils. The vast majority of studies on witness testimony deal with eyewitness accounts (e.g., [12]) and a few with earwitness testimony (e.g., [13]). To our knowledge, the possibility of identifying individuals in a forensic setting by way of odor, henceforth referred to as *nosewitness identification*, has been completely overlooked. A central and debated problem of eyewitness studies concerns the effect of the emotions experienced while witnessing a crime. Although it seems evolutionarily relevant that highly arousing events (e.g., witnessing a crime) would augment our recollection of relevant details [14], studies on eyewitness identification typically show a decline in performance for actual recognition of the culprit [15, 16]. However, few if any studies have looked at recognition memory for odors encoded during negative emotions.

With this background, we set out in Experiment 1 to define and test an experimental model of nosewitness identification in order to investigate human olfactory memory for body odor in a forensic setting, including emotion-evoking crimes. A second experiment expanded on this issue by testing the memory effect of negative emotion in the nosewitness situation using a conventional lineup identification test.

## Experiment 1

In Experiment 1, we probed olfactory identification in a target-present, forced-choice, lineup memory test for *nosewitness* and *neutral* conditions. In the nosewitness condition, an authentic video of a violent crime was presented along with a body odor. Written instructions aimed to get the participant in the mindset of a witness to make the experimental model of the nosewitness situation more realistic. In the neutral condition, an emotionally neutral video was presented along with a body odor and neutral instructions were used. In both conditions, the cover story stated that the body odor was coming from the male character (culprit). In a later lineup test, participants got to decide which body odor of five was the culprit’s. The aim was to assess whether nosewitness identification would perform better than the neutral condition and thus be a possible identification method.

### Materials and Methods

Both experiments were approved by the Ethics Committee of the University of Aveiro, Portugal. Moreover, the guidelines of the Declaration of Helsinki and the standards of the American Psychological Association were followed.

**Body odor samples.** Body odor (BO) samples were collected from the armpits of 20 healthy male students, aged between 18 and 39 years (*M* = 24.70, *SD* = 6.15), while they worked on a non-stressful assignment over a period of 2h30minutes. The donors refrained from using products and performing activities that would alter their natural BO before and during the sampling. These exogenous BO components may be a part of the body odor in real life and their influence should be systematically investigated in the future. The BO samples were frozen and thawed before testing the same number of times in each condition (see S1 File).

**Participants.** Eighty students (20 men and 20 women in each condition) from the University of Aveiro, Portugal, aged between 18 and 49 years (*M* = 23.19; *SD* = 5.68), volunteered to participate. The lack of available tools providing representative scores for olfactory identification skills in the Portuguese population prevented us from providing standardized olfactory identification scores. However, in order to identify variables possibly confounding olfactory identification performance, participants were asked to indicate any olfactory problems or other conditions that may have influenced their olfactory identification abilities (e.g., nasal obstruction). Moreover, the participants did not suffer from any mental, neurological, or metabolic diseases, and were medication free. They were asked to refrain from eating (e.g., gum), drinking coffee, or using any products that could interfere with their ability to smell one hour before testing. Participants and donors signed a written informed consent form and were rewarded with either course credits or 5 euros.

**Design and procedure.** Participants were randomly assigned to one of the two conditions (nosewitness and neutral) in which they viewed a one-minute audio-visual presentation (video clip) of an event involving a man (culprit) and a woman. Instructions (see S1 File) were displayed on the screen for 30 seconds

A body odor was presented continuously during the video clip in wide-mouth glass jars, which the participants held under their noses with their dominant hands. Participants were instructed to breathe through their noses.

In a 15-minute period between the video clip (witness session) and lineup test, participants rated the video in terms of vividness, pleasantness and arousal (see S1 File), and completed a questionnaire assessing trait anxiety (STAI-T, [17]).

In the lineup test, participants were instructed to identify the odor of the culprit whose BO they smelled during the video presentation. Participants chose from a lineup of five BO samples—the culprit BO and four foil BOs. This five-alternative, forced-choice, target-present procedure was chosen in order to obtain a high power and bias-free measure of the identification performance. BO samples were presented in wide-mouth glass jars, from left to right, with no time restriction to smell the BO, but without the chance to resample previous BOs. The position of the BOs in the lineup was counterbalanced so that it was presented in each of the five positions the same number of times, and either as culprit or foils (see S1 File). To balance constraints of odor adaptation and working memory capacity [18], an interstimulus-interval of 6 seconds was chosen. After the identification response, participants were asked to rate their confidence in their decision on a scale from 0 to 100%. Participants were also asked to rate each BO sample’s perceived intensity (1–9), pleasantness (1–9), and familiarity (1–9) (for results, see S1 File, Experiment 1). Finally, participants were thanked and informed about the nature of the experiment.

Both before and after the task, participants rated their perceived stress using a Visual Analogue Scale (VAS) and their state anxiety levels (STAI-S [17]). The purpose was to monitor whether participants were in distress when they left the lab (they were not), as well as to assess whether any of these measures were correlated with performance (see S1 File).

### Results and Discussion

**Figure 1:**
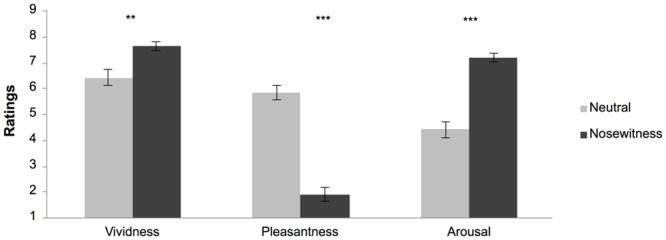
Means (SE) of subjective ratings of the videos in the neutral and nosewitness conditions on a 9-point rating scale in Experiment 1. Note that the pleasantness scale is bipolar with 5 as neutral and with higher and lower extremes reflecting high pleasantness and high unpleasantness, respectively. ** p < .01, *** p <.001.

**Nosewitness experience.** Independent samples *t-*tests of ratings were run in order to assess whether the crime and neutral videos were indeed perceived as different. The results confirmed that crime videos were rated as significantly more arousing (*t*(78) = -7.56, *p* < .0001, *d* = -1.71), more vivid (*t*(78) = -3.32, *p* < .01, *d* = -0.75), and more unpleasant (*t*(78) = 10.41, *p* < .0001, *d* = 2.36) than the neutral videos (Fig. 1).

**Figure 2:**
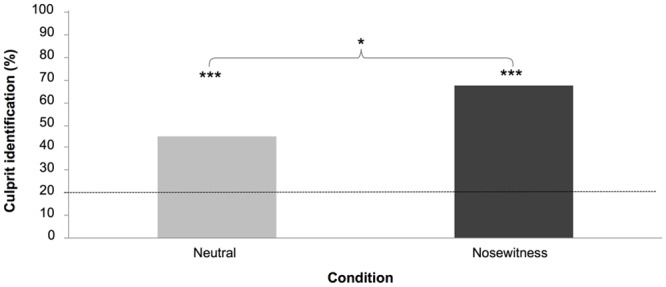
Percentage of participants correctly identifying the culprit odor in the neutral and nosewitness condition in Experiment 1. Performance levels of conditions differ from chance and each other. Dashed line represents chance performance. *p < .05; difference between conditions (nosewitness vs. neutral). ***p < .0001; binomial probabilities indicting that the performance (in each condition) is significantly above chance.

**Lineup test identification.** Binomial probabilities for the number of correct responses were calculated (Fig. 2). Performance for the neutral condition (18 correct responses = 45%, binomial probability, *p*< .001) and the nosewitness condition (27 correct responses = 68%, binomial probability, *p*< .001) was above chance level (8 correct responses = 20%). Chi-square tests based on counts of participants (being correct or not) were used to analyze the differences in correctness of culprit identification between the conditions. Participants in the nosewitness condition were significantly better compared to participants in the neutral video condition (68% vs. 45%, respectively; χ^2^ (1) = 4.11; *p* < .05; Cramér’s φ =.23).

Overall, the results indicate that participants could identify the body odor associated with a culprit well above chance. This was particularly evident under nosewitness conditions (see S1 File, for analyses of sex differences).

**Confidence of identification.** The results also showed that accuracy of culprit identification was not reliably correlated with participants’ confidence in their identification for the nosewitness condition (*r_pb_* (38) = -.03, p = .85) and the neutral witness condition (*r_pb_* (38) = .13, *p* = .44). Therefore, and in line with the eyewitness identification literature (e.g., [19]), confidence was not a good predictor of accuracy in BO identification performance. Although there is some face validity in the notion that confident witnesses should be more accurate, moderate correlations have questioned this, as well as the value of confidence in the assessment of eyewitness accuracy (e.g., [20]).

## Experiment 2

The higher emotional ratings of the crime videos and the higher identification accuracy in the nosewitness condition in Experiment 1 suggest that negative emotion during encoding of body odors may be what has boosted recognition. As noted above, this would be in opposition to what is typically found in the literature for eyewitness identification, namely an impaired recognition of the culprit [15, 16].

The instructions of Experiment 1, which aimed to involve the participants in the nosewitness condition in the mindset of a witness (see S1 File), may have led them to encode the body odor more intentionally, thus leading to a higher performance. Experiment 2 targeted the hypothesis that emotion would boost nosewitness performance by only varying the video type between conditions and having identical instructions for the crime and neutral videos. In the current experiment, we also used a conventional witness identification design involving culprit-present and culprit-absent lineups and a double-blind procedure to accurately assess both performance levels and decision bias (for a review, see [21]).

### Materials and Methods

**Body odor samples.** BO samples were collected from the armpits of 20 healthy male students, aged between 18 and 24 years (M = 20.40, SD = 1.85), following the same procedure as in Experiment 1, but over a longer sampling period of 4h (see S1 File). The donors refrained from using products and performing activities that would alter their natural BO before and during the sampling. The BO samples were frozen and thawed before testing (see S1 File).

To ensure that nosewitness performance was not hampered by too-weak BOs or driven by too extreme BOs, samples were carefully selected according to six rating scales in a pilot experiment (see S1 File).

**Participants.** Eighty students (41 women, 39 men) from the University of Aveiro, Portugal, aged between 17 and 38 years (*M* = 21.96; *SD* = 4.64), volunteered to participate.

The participants did not suffer from any mental, neurological, metabolic, or respiratory diseases, and were medication free. They were asked to refrain from eating (e.g., gum), drinking coffee, or using any products that could interfere with their ability to smell one hour before testing. Participants and donors signed a written informed consent form and were rewarded with either course credits or 5 euros.

**Design and procedure.** The participants were randomized into the nosewitness and neutral conditions (40 in each) and target-present (20 men and 20 women) and target-absent (19 men and 21 women) lineups. The material (videos and scales), inclusion criteria, behavioral restrictions, and rewards were the same as those used in Experiment 1.

Participants rated the videos using a 100 mm VAS for vividness, emotional arousal, and pleasantness, from *not at all* to *very much*. The instructions were displayed on the screen for 15 seconds.

In the lineup, we used target-present (TP) trials consisting of one culprit BO and four foils (position for culprit BO was balanced) and target-absent (TA) trials consisting of five foils. Unbiased instructions were given to the participants, who were told that the culprit might or might not be present in the lineup ([22]; see S1 File). The lineup administrator was blind to the presence/absence and position of the culprit BO (e.g., [23]). In both nosewitness and neutral conditions, 20 individuals were presented with target-present lineups and 20 with target-absent lineups. After reporting whether a culprit was present or absent in the lineup, participants rated their level of confidence.

BO samples were presented in wide-mouth glass jars from left to right, with no time restriction to smell the BO, but without the chance to resample previous BOs. Although the procedure was similar to that of Experiment 1, i.e., the position of the BOs was counterbalanced so that they were presented in each of the five positions in the lineup the same number of times, in this experiment BOs were not used as either targets or foils, since two of the conditions involved target-absent trials (nosewitness target-absent; neutral target-absent). Moreover, in the target-absent conditions of Experiment 2, six BOs were needed, instead of five as in Experiment 1 (one BO during encoding, and five different BOs in the lineup) (see S1 File). After the identification response, participants were asked to rate their confidence in their decision on a scale from 0 to 100%. Finally, participants were thanked and informed about the real nature of the experiment.

Both before and after the task, participants rated their perceived stress using a Visual Analogue Scale (VAS) and their state anxiety levels (STAI-S, [17]). The purpose was to monitor whether participants were in distress when they left the lab (they were not), as well as to assess whether any of these measures were correlated with performance (see S1 File).

### Results and Discussion

**Figure 3:**
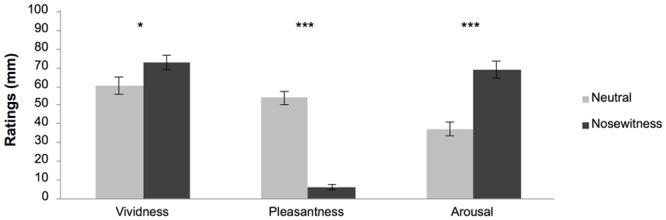
Means (SE) of subjective ratings of the videos in the neutral and nosewitness conditions on a 100mm Visual Analogue Scale in Experiment 2. Note that the pleasantness scale is bipolar with 50 as neutral and with higher and lower extremes reflecting high pleasantness and high unpleasantness, respectively. * p ≤ .05, *** p< .001.

**Nosewitness experience.** Independent samples t-tests of ratings were run in order to assess whether the crime and neutral videos were indeed perceived as different. As in Experiment 1, the results confirmed that crime videos were rated as significantly more arousing (t (78) = -5.38, *p* < .0001, d = -1.22), more vivid (t (78) = -1.96, *p* = .05, d = -0.44), and more unpleasant (t (78) = 11.71, *p* < .0001, d = 2.65) than the neutral videos (see Fig. 3).

**Lineup identification.** Separate analyses were performed for TP and TA lineups. Responses for TP were coded as either Hit (correct identification), False-Positive (identification of non-culprit BO), or Miss (failure to identify the culprit). For TA, responses were coded as Correct Rejection (correct rejection of all in the lineup) and False-Positive (incorrect identification of a culprit).

For the TP trials, a chi-square was run to test the difference in lineup identification performance between the nosewitness and neutral conditions, with the results yielding a statistically significant difference (χ^2^ (2) = 6.51; *p* = .04, Cramér’s φ = .40).

**Figure 4:**
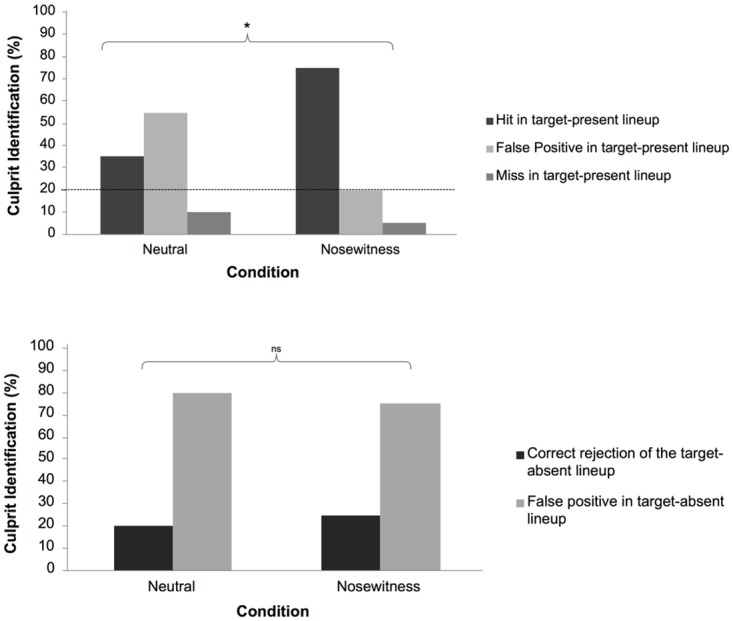
Percentage of participants correctly identifying the culprit odor in Target-Present lineups and percentage of correct rejections and false-positives in Target-Absent lineups in the neutral and nosewitness conditions in Experiment 2. * p < .05.

As shown in Fig. 4, participants in the nosewitness condition showed higher accuracy in identifying the culprit BO (hits, 75%) compared to the neutral condition (35%). Moreover, participants in the nosewitness condition were less likely to identify an innocent foil (false positive, 20%) compared to participants in the neutral condition (55%). Finally, participants in the nosewitness condition were also less likely to claim that culprit BO was not present in the lineup compared with participants in the neutral condition (miss, 5% and 10%, respectively).

For TA trials (Fig. 4), an additional chi-square was run. However, there was no significant difference between the neutral and the nosewitness conditions (χ^2^ (1) = .14; *p* = .71; Cramér’s φ = .06 (Fig. 4). Although the positive effect of emotional videos on nosewitness performance runs counter to what is typically observed in eyewitness studies, the current results do concur with studies on eyewitness testimony in that stress has a larger effect on TP trials than TA trials [15, 16].

**Confidence of identification.** In line with Experiment 1, the results also showed that accuracy of culprit identification was not reliably correlated with participants’ confidence in their identification for the nosewitness condition (*r_pb_* (38) = .158, *p* = .330) and the neutral witness condition (*r_pb_* (38) = .159, *p* = .326). These correlations were also insignificant for target-present lineups (*r_pb_* (38) = .11, p = .52) and target-absent lineups (*r_pb_* (38) = -.01, *p* = .93). Thus, confidence was not a good predictor of accuracy in BO identification performance.

## General Discussion

In two studies, we explored nosewitness identification following violent and emotional videos of crimes. In parallel to what has been shown in eye- and earwitness studies (reviewed in [21] and [13], respectively), our results suggest that humans are capable of identifying a culprit by way of odor. Interestingly, olfactory lineup identification performance was significantly better following emotional videos compared to neutral ones. Although there is much evidence that emotion can enhance aspects of eyewitness memory for central information to the detriment of peripheral information [24], a meta-analysis of eyewitness studies shows a decline in lineup face identification performance as a function of the levels of stress [15, 16]. Yet, in the current study emotional processing of body odor at encoding seems to explain the superior performance in the nosewitness condition, compared to the neutral condition.

Odors and faces are inherently different types of stimuli in several ways [25], and memory for odors may thus be differently influenced by emotion than memory for faces. Although the relation between emotion and olfaction has gained considerable interest, the current study is the first, to our knowledge, indicating that negative emotion during encoding can enhance actual recognition. However, there is much to do in order to complete the picture, such as direct comparative studies of odor and visual stimuli. In the current study, the observations that memory for odors encoded during negative, compared to neutral, emotion enhanced performance may relate to the functional neuroanatomy of olfaction. The odor signal projects directly from the olfactory bulb to emotional parts of the brain, such as the amygdala, and olfactory functioning in general has been shown to be highly dependent on the emotional state [26]. In line with this, heightened emotion during encoding of visual stimuli induced by an ambient odor enhances the effectiveness of the odor as a cue to visual memory [27]. Memories evoked by odors are also perceived as more emotional compared to memories evoked by other sensory modalities [28]. Interestingly, exposure to the body odor of unknown individuals heightens activity in areas of the human brain’s threat circuitry—the amygdala and insula—relative to the body odor of familiar persons [29].

The results in Experiment 2 also indicated a superiority of the lineup identification for target-present trials compared to target-absent trials, which is consistent with the notion that olfactory cognition is especially prone to false alarms. It has been argued that olfactory cognition is set to overestimate stimulation for the purpose of serving as a warning system with safe and liberal decision criteria for detection [30]. In parallel, liberal decision criteria (i.e., high incidence of false alarms) for short-term recognition of common object odors have also been reported [31].

When the perpetrator and the victim are close to one another, as in crimes of sexual and physical assault, and especially under visually obscure conditions, an olfactory cue may be the prevailing detail for later recognition [32–34]. In fact, the *Cognitive Interview* (a method for interviewing eyewitnesses and victims about what they remember from a crime scene) instructions encourage witnesses to remember stimuli from every sensory modality, including odors [35]. In the present study, participants were also made aware of the olfactory stimulation. However, in real life situations, victims will most likely process olfactory information without awareness [36]. Future studies should investigate whether nosewitness performance is maintained in implicit memory tests.

For nosewitness lineup tests to become forensically useful a number of practical problems must be solved, including those concerning the sampling of a suspect’s body odor and the choice of foils. However, the experimental model for nosewitness studies presented here paves the way for future studies investigating the interplay between emotion and olfaction and the possible use of the sense of smell in forensic settings.

## Supporting Information

S1 File Nosewitness Identification: Effects of Negative Emotion. (DOCX)

## References

[1] Havlicek J, Lenochova P (2006) The effect of meat consumption on body odor attractiveness. Chem Senses 31: 747–752. Available: http://www.ncbi.nlm.nih.gov/pubmed/16891352. Accessed 2014 May 28 10.1093/chemse/bjl017 16891352

[2] Mitro S, Gordon AR, Olsson MJ, Lundström JN (2012) The smell of age: perception and discrimination of body odors of different ages. PLoS One 7: e38110 Available: http://www.pubmedcentral.nih.gov/articlerender.fcgi?artid=3364187&tool=pmcentrez&rendertype=abstract. Accessed 2014 April 6 10.1371/journal.pone.0038110 22666457 PMC3364187

[3] Doty RL, Ford M, Preti G, Huggins GR (1975) Changes in the intensity and pleasantness of human vaginal odors during the menstrual cycle. Science (80- ) 190: 1316–1318. 10.1126/science.1239080 1239080

[4] Olsson MJ, Lundström JN, Kimball BA, Gordon AR, Karshikoff B, et al (2014) The scent of disease: human body odor contains an early chemosensory cue of sickness. Psychol Sci 25: 817–823. Available: http://www.ncbi.nlm.nih.gov/pubmed/24452606. Accessed 2014 May 28 10.1177/0956797613515681 24452606

[5] Eggert F, Luszyk D, Haberkorn K, Wobst B, Vostrowsky O, et al (1999) The major histocompatibility complex and the chemosensory signalling of individuality in humans. Genetica 104: 265–273. Available: http://www.ncbi.nlm.nih.gov/pubmed/10386393 10.1023/A:1026431303879 10386393

[6] Rodriguez-Lujan I, Bailador G, Sanchez-Avila C, Herrero A, Vidal-de-Miguel G (2013) Analysis of pattern recognition and dimensionality reduction techniques for odor biometrics. Knowledge-Based Syst 52: 279–289. Available: http://linkinghub.elsevier.com/retrieve/pii/S0950705113002323. Accessed 2014 May 29 10.1016/j.knosys.2013.08.002

[7] Roberts SC, Gosling LM, Spector TD, Miller P, Penn DJ, et al (2005) Body odor similarity in noncohabiting twins. Chem Senses 30: 651–656. Available: http://www.ncbi.nlm.nih.gov/pubmed/16162644. Accessed 2014 May 28 10.1093/chemse/bji058 16162644

[8] Pinc L, Bartoš L, Reslová A, Kotrba R (2011) Dogs discriminate identical twins. PLoS One 6: e20704 Available: http://www.pubmedcentral.nih.gov/articlerender.fcgi?artid=3115944&tool=pmcentrez&rendertype=abstract. Accessed 2014 Mar 26 10.1371/journal.pone.0020704 21698282 PMC3115944

[9] Schoon GAA (1996) Scent identification lineups by dogs ( Cunis familiaris ) : experimental design and forensic application. Appl Anim Behav Sci 49: 257–267. 10.1016/0168-1591(95)00656-7

[10] Schoon GAA (2005) The effect of the ageing of crime scene objects on the results of scent identification line-ups using trained dogs. Forensic Sci Int 147: 43–47. Available: http://www.ncbi.nlm.nih.gov/pubmed/15541591. Accessed 2014 Mar 24 10.1016/j.forsciint.2004.04.080 15541591

[11] Prada PA, Furton KG (2008) Human scent detection: A Review of its developments and forensic applications. Rev Ciências Forenses 1: 81–87.

[12] Clark SE, Howell RT, Davey SL (2008) Regularities in eyewitness identification. Law Hum Behav 32: 187–218. Available: http://www.ncbi.nlm.nih.gov/pubmed/17410411. Accessed 2014 May 16 10.1007/s10979-006-9082-4 17410411

[13] Hollien H (2012) On earwitness lineups. Investig Sci J 4: 1–17.

[14] Kensinger EA (2007) Negative emotion enhances memory accuracy behavioral and neuroimaging evidence. Curr Dir Psychol Sci 16: 213–218. 10.1111/j.1467-8721.2007.00506.x

[15] Deffenbacher KA, Bornstein BH, Penrod SD, McGorty EK (2004) A Meta-Analytic Review of the Effects of High Stress on Eyewitness Memory. Law Hum Behav 28: 687–706. Available: http://doi.apa.org/getdoi.cfm?doi=10.1007/s10979–004–0565-x. Accessed 2014 Mar 26 10.1007/s10979-004-0565-x 15732653

[16] Houston KA, Clifford BR, Phillips LH, Memon A (2013) The emotional eyewitness: the effects of emotion on specific aspects of eyewitness recall and recognition performance. Emotion 13: 118–128. Available: http://www.ncbi.nlm.nih.gov/pubmed/22775133. Accessed 2014 May 26 10.1037/a0029220 22775133

[17] Spielberger CD (1983) Manual for the State-Trait Anxiety Inventory STAI (Form Y). Palo Alto: Consulting Psychologists Press.

[18] Jönsson FU, Møller P, Olsson MJ (2011) Olfactory working memory: effects of verbalization on the 2-back task. Mem Cognit 39: 1023–1032. Available: http://www.ncbi.nlm.nih.gov/pubmed/21369969. Accessed 2014 May 29 10.3758/s13421-011-0080-5 21369969

[19] Krug K (2007) The relationship between confidence and accuracy: current thoughts of the literature and a new area of research. Appl Psychol Crim Justice 3: 7–41.

[20] Sporer SL, Penrod S, Read D, Cutler B (1995) No TitleChoosing, confidence, and accuracy: A meta-analysis of the confidence-accuracy relation in eyewitness identification studies. Psychol Bull 118: 315–327. 10.1037/0033-2909.118.3.315

[21] Leach AM, Cutler BL, Van Wallendael L (2009) Lineups and Eyewitness Identification. Annu Rev Law Soc Sci 5: 157–178. Available: http://www.annualreviews.org/doi/abs/10.1146/annurev.lawsocsci.093008.131529. Accessed 2014 Apr 7 10.1146/annurev.lawsocsci.093008.131529

[22] Wells GL, Small M, Penrod S, Malpass RS, Fulero SM, et al (1998) Eyewitness identification procedures : recommendations for lineups and photospreads. Law Hum Behav 22: 1–39. 10.1023/A:1025750605807

[23] Rodriguez DN, Berry MA (2013) Eyewitness Science and the Call for Double-Blind Lineup Administration. J Criminol 2013: 1–10. Available: http://www.hindawi.com/journals/jcrim/2013/530523/ 10.1155/2013/530523

[24] Easterbrook JA (1959) The effect of emotion on cue utilization and the organization of behavior. Psychol Rev 66: 183–201. 10.1037/h0047707. 13658305

[25] Stevenson RJ (2014) Object Concepts in the Chemical Senses. Cogn Sci: 1–24. Available: http://www.ncbi.nlm.nih.gov/pubmed/24641582. Accessed 2014 June 30 10.1111/cogs.12111 24641582

[26] Krusemark EA, Novak LR, Gitelman DR, Li W (2013) When the sense of smell meets emotion: anxiety-state-dependent olfactory processing and neural circuitry adaptation. J Neurosci 33: 15324–15332. Available: http://www.pubmedcentral.nih.gov/articlerender.fcgi?artid=3782615&tool=pmcentrez&rendertype=abstract. Accessed 2014 May 28 10.1523/JNEUROSCI.1835-13.2013 24068799 PMC3782615

[27] Herz RS (1997) Emotion experienced during encoding enhances odor retrieval cue effectiveness. Am J Psychol 110: 489–505. Available: http://www.ncbi.nlm.nih.gov/pubmed/9479745 10.2307/1423407 9479745

[28] Herz RS (2004) A Naturalistic Analysis of Autobiographical Memories Triggered by Olfactory Visual and Auditory Stimuli. Chem Senses 29: 217–224. Available: http://www.chemse.oupjournals.org/cgi/doi/10.1093/chemse/bjh025. Accessed 2014 May 28 10.1093/chemse/bjh025 15047596

[29] Lundström JN, Boyle JA, Zatorre RJ, Jones-Gotman M (2008) Functional neuronal processing of body odors differs from that of similar common odors. Cereb Cortex 18: 1466–1474. Available: http://www.ncbi.nlm.nih.gov/pubmed/17934190. Accessed 2014 May 29 10.1093/cercor/bhm178 17934190

[30] Engen T (1991) Odor sensation and memory. New York, NY: Praeger Publishers.

[31] Olsson MJ, Lundgren EB, Soares SC, Johansson M (2009) Odor Memory Performance and Memory Awareness: A Comparison to Word Memory Across Orienting Tasks and Retention Intervals. Chemosens Percept 2: 161–171. Available: http://link.springer.com/10.1007/s12078–009–9051–7. Accessed 2014 Mar 24 10.1007/s12078-009-9051-7

[32] Christianson SÅ (1992) Remembering emotional events: Potential mechanism. In: Christianson S-Å, editor. Handbook of emotion and memory: Research and theory. New Jersey: LEA.

[33] Doege D (1992) Rape victim remembers fear, smell, feel of attack. Milwaukee Sentin: 9 Available: http://news.google.com/newspapers?nid=1368&dat=19921119&id=JYVQAAAAIBAJ&sjid=8BIEAAAAIBAJ&pg=6901,4929216.

[34] LGIT (2011) LGIT’s roll call reporter. Local Govenment Insur Trust. Available: http://md-lgit.civicplus.com/DocumentCenter/Home/View/671.

[35] Brunel M, Py J, Launay C (2013) Cost and benefit of a new instruction for the cognitive interview: the open depth instruction. Psychol Crime Law 19: 845–863. Available: http://www.tandfonline.com/doi/abs/10.1080/1068316X.2012.684058. Accessed 2014 June 24 10.1080/1068316X.2012.684058

[36] Degel J, Köster EP (1999) Odors: implicit memory and performance effects. Chem Senses 24: 317–325. Available: http://www.ncbi.nlm.nih.gov/pubmed/10400450 10.1093/chemse/24.3.317 10400450

