# Correction: Nosewitness Identification: Effects of Negative Emotion

**DOI:** 10.1371/journal.pone.0121012

**Published:** 2015-03-16

**Authors:** The PLOS ONE Staff

The following information is missing from the Funding section: Portuguese Foundation of Science and Technology (FCT) (PTDC/MHC-PCN/4842/2012 to SCS).
