# Supplementary material for: Nosewitness Identification: Effects of Negative Emotion
Source: PLoS One. 2015 Jan 22;10(1):e0116706. doi: 10.1371/journal.pone.0116706 (PMC4303424; doi:10.1371/journal.pone.0116706)
Supplement: S1 File — (DOCX) [file pone.0116706.s001.docx]

**Supporting Information S1**

**Nosewitness Identification: Effects of Negative Emotion**

Laura Alho^1,2*^, Sandra C. Soares^1,2,3*^, Jacqueline Ferreira^1,2^, Marta Rocha^1^, Carlos F. Silva^1,2,3^, & Mats J. Olsson^4^

^1^Department of Education, University of Aveiro, Aveiro, Portugal

^2^Institute for Biomedical Imaging and Life Sciences (IBILI), Faculty of Medicine, University of Coimbra, Coimbra, Portugal

^3^Center for Health Technology and Services Research (CINTESIS), Faculty of Medicine, University of Oporto, Portugal

^4^Department of Clinical Neuroscience, Division for Psychology, Karolinska Institutet, Stockholm, Sweden

*Both authors contributed equally to the study.

Correspondence concerning this article should be addressed to Sandra C. Soares, University of Aveiro, Department of Education, Campus Universitário de Santiago, 3810-193 Aveiro, Portugal. Email: [sandra.soares@ua.pt](mailto:sandra.soares@ua.pt)

**Experiment 1**

**Body Odor Samples**

Twenty healthy students from the University of Aveiro, Portugal, donated body odor (BO) samples. Eligible donors were male (as the vast majority of criminals are men [1]), non-smoking, medication free, and had no physical, metabolic or mental disease. In addition, donors were instructed to refrain from using fragrant hygiene products, drinking alcohol, and eating spicy foods in order to avoid altering their natural BO. Donors signed an informed consent form and were rewarded either with course credits or 5 euros.

BO was collected on nursing pads (Mimo Natura, Portugal) sewn into the armpits of t-shirts previously washed with odorless detergent (e.g., [2]). Participants wore the t-shirts in a campus lecture room for 2h30m. To standardize their activity, they were asked to write a letter introducing their university to an imagined incoming exchange student. The nursing pads were then collected from each armpit, divided into equal size quadrants, stored in closed zip-locked bags, and frozen at -20°C.

The pad quadrants were thawed 1 hour before testing. Two pad quadrants were placed along the walls of wide-mouthed glass jars with lids and were used as BO samples (see below). To prevent contamination, odor samples were always handled with surgical gloves.

**Counterbalancing of Body Odors in Experiment 1**

In the Experiment 1 we had four lineups, each one with five BOs from 5 different donors (for exemplification purposes, assume that each letter A-E corresponds to a different body odor, and that the letter “A” corresponds to the culprit’s BO: A-B-C-D-E). We had 40 participants in each condition (*nosewitness* and neutral conditions), with sets of five participants, each exposed to one lineup with one culprit BO and four foils, with the all the positions counterbalanced. Therefore, participant #1 was exposed to the lineup A-B-C-D-E, participant #2 was exposed to the lineup E-A-B-C-D, participant #3 to D-E-A-B-C, participant #4 to C-D-E-A-B, and participant #5 to B-C-D-E-A. Thus, the culprit BO and the foils were presented in different positions to each participant. Each set of five participants was exposed to a lineup with different body odors. The culprit BOs used in the lineups were also used as foils, and the same BO lineups were used in the neutral condition in order to assure that any differences in performance were not due to differences in the BOs presented.

**Nosewitness and neutral videos for Experiment 1**

In the nosewitness condition, participants either witnessed a homicide, domestic violence, a kidnapping, a sexual assault, or a theft with a hostage situation. In the neutral witness condition, daily life scenes were presented: either a walk in the park, a walk on the beach, a man teaching a woman how to fish, an interview of an artist in a gallery, or a photography team working in an historical city. All the videos captured a man and a woman in the scene, and were presented on a 17” computer screen with headphones. To characterize the 10 videos, they were rated for vividness and emotional arousal on a scale from 1 (none) to 9 (very much), and rated for pleasantness on a scale from 1(very unpleasant) to 9 (very pleasant) with 5 indicating neutral.

**Instructions given to participants before the presentation of the video**

For the nosewitness condition the instructions were the following: “In crime situations odor may be a cue that can help to perform a positive identification of the perpetrator. You will now see a real crime captured by a video camera. During the video you will be exposed to an odor collected from the perpetrator of the crime you will be witnessing”.

For the neutral condition the instructions were the following: “Odors are present in every domain of our life, including in food, clothes, perfumes, and so on. You will now see a real situation, captured by a video camera. During the video you will be exposed to an odor collected from the man presented in the video”.

**Instructions given to participants when the lineup was presented**

“You have a lineup with five different body odors in front of you and you will smell all of them from left to right. The body odor that you smelled during the video is in this lineup. After you smell all of the BOs, fill your response sheet, please.”

**Perceptual ratings of body odors**

In addition to the subjective evaluation of the films, participants were also asked to rate the different body odors presented in the lineup. Perceptual ratings of the culprit odor and the foils were analyzed using separate independent samples t-tests for intensity, pleasantness, and familiarity. It should however be noted that these ratings were performed after the lineup identification to avoid interfering with the identification procedure. In a comparison between conditions, the culprit BO was on average perceived as more unpleasant in the nosewitness condition than in the neutral condition (*t* (78) = 2.10, *p* < .05, d = 0.48). This was, however, also true for the average foil (*t* (78) = 2.56, *p* < .05, d = 0.58). No such statistical differences were found for the ratings of intensity and familiarity.

**Participants’ stress and anxiety levels**

Prior to the presentation of each video, participants rated their perceived stress on a 100 mm visual analogue scale (VAS), as well as their state anxiety using STAI-S [3]. The stress measurement was repeated after the lineup test. Paired t-tests were performed within conditions to compare the stress levels before and after the task. In the nosewitness condition, the stress levels of the participants decreased significantly (*t* (39) = 5.65, *p* < .001, d = 1.83; from *M* = 47.45, *SD* = 25.49, to *M* = 23.40, *SD* = 17.48), whereas in the neutral condition the stress levels slightly decreased (*t* (39) = .80, *p* = .43, d = 0.26; from *M* = 23.10, *SD* = 21.30. to *M* = 21.40, *SD* = 19.58). However, the overall stress levels of the participants were low and they did not leave the lab in distress.

In the 15-min delay between the witness session and lineup test, participants completed a questionnaire assessing trait anxiety (STAI-T, [3]). Neither trait nor state anxiety correlated with later identification performance (*r_pb_* (78) = -.12, *p* = .27 and *r_pb_* (78) = .02, *p* = .89, respectively).

**Sex differences**

As women are typically victims of assault perpetrated by men, we intended to further investigate whether exposing women to male body odors resulted in differences in performance between conditions. Although the chi-square tests did not show statistically significant differences between women and men for the nosewitness and neutral conditions (*p*s > .05), women had nominally higher performances in the nosewitness condition (15 hits, corresponding to 56%) compared to men (12 hits, corresponding to 44%). Moreover, in the neutral condition no difference was found between women and men (9 hits, 50% for each sex).

**Experiment 2**

**Body Odor Samples**

All the sampling procedures were the same as in Experiment 1. The only difference was the duration of the sampling: participants wore the t-shirts for 4h at the university campus.

**Pre-evaluation of the BOs**

To standardize the lineups and present BOs that did not differ in terms of hedonic characteristics, two sessions of a pre-evaluation of the BOs were made. Twenty individuals (10 men and 10 women, half in each session) rated the BO samples in a 100mm Visual Analogue Scale (VAS), ranging from *not at all* to *very much so*, in terms of pleasantness, familiarity, intensity, attractiveness, arousal, and distinctiveness. Following Z-score transformation, the samples of two donors were excluded since they differed ± 2 SD from the group mean in at least one of the characteristics.

**Counterbalancing of Body Odors in Experiment 2**

In Experiment 2, we used the same procedure of counterbalancing the position of all the BOs as in Experiment 1. However, this time the targets were not used as foils to ensure that any differences between conditions were not due to the presentation of different body odors since in Experiment 2 we also included target absent trials (i.e., without the culprit BO). Therefore, we used different targets in each set of 5 participants, in addition to using and thawing the samples the same number of times in order to prevent bacterial degradation. In the target-absent condition, we used 6 odors instead of five (one culprit BO during the visualization of the video-clip, and 5 different BOs in the lineup, where the culprit BO was not present).

**Nosewitness and neutral videos**

The videos were the same as those used in Experiment 1.

**Instructions given to participants before the presentation of the video**

For the nosewitness condition the instructions were the following: “You will see a real crime captured by a video camera. During the video you will be exposed to an odor collected from the perpetrator of the crime you will be watching”.

For the neutral condition the instructions were the following: “You will see a real situation captured by a video camera. During the video you will be exposed to an odor collected from the man presented in the video that you will be watching”.

**Instructions given to participants when the lineup was presented**

“You have a lineup with five different body odors in front of you and you will smell all of them from left to right. The body odor that you smelled during the video might or might not be present in the lineup”.

**Participants’ stress and anxiety levels**

Prior to the presentation of each video, participants rated their perceived stress on a 100 mm VAS, as well as their state anxiety using STAI-S [3]. Both of the measurements were repeated after the lineup test. Paired t-tests were performed for the stress measurement for each condition. Results showed no significant differences for the nosewitness condition from the first to second measurement (*t* (39) = -1.57, *p* = .13, d = -.50; *M* = 24.23, *SD* = 18.96; *M* = 29.08, *SD* = 24.06, respectively) or for the neutral condition (*t* (39) = 1.60, *p* = .12, d = .51; *M* = 26.23, *SD* = 22.12; *M* = 21.90, *SD* = 20.97, respectively). As in Experiment 1, the overall stress levels of the participants were low and they did not leave the lab in distress.

Paired t-tests were also performed for state anxiety within conditions (nosewitness vs. neutral). The results showed that there were no significant differences from the first to second measurement for the nosewitness condition (*t* (39) = -1.47, *p* = .15, d = -.47; *M* = 31.73, *SD* = 5.84; *M* = 33.23, *SD* = 8.37, respectively) or for the neutral condition (*t* (39) =-.03, *p* = .98, d = -.009; *M* = 31.68, *SD* = 8.64; *M* = 31.70, *SD* = 7.99, respectively).

In the 15-min delay between the witness session and lineup test, participants completed the STAI-T (trait anxiety). Neither trait nor state anxiety correlated with later identification performance (*r_pb_* (78) = -.12, *p* = .29 and *r_pb_* (78) = .05, *p* = .66, respectively).

**Emotion rating scales**

During the 15-minute delay, and in line with Houston’s study [4], participants rated their experienced level of irritation, annoyance, outrage, anger, happiness, sadness, sympathy, disgust, upset, fright, anxiety, and relief using a 100 mm VAS (ranging from *not at all* to *very much so*). Table S1 shows the perceived differences in emotions by the participants in each condition. For all the emotions, except annoyance and relief (both *ps* > .05), the results showed a statistically significant difference between the nosewitness and neutral conditions (all *ps* < .05), indicating considerably stronger negative emotion in the nosewitness condition.

| **Emotion** | **Condition Group** | **N** | **Mean** | **Std. Deviation** |
| --- | --- | --- | --- | --- |
| Irritation | Neutral | 40 | 6.3000 | 16.46628 |
|  | Nosewitness | 40 | 44.3250 | 28.29006 |
| Annoyance | Neutral | 40 | 23.2250 | 29.30694 |
|  | Nosewitness | 40 | 20.9500 | 20.00250 |
| Outrage | Neutral | 40 | 5.2250 | 12.10952 |
|  | Nosewitness | 40 | 37.6250 | 31.23418 |
| Anger | Neutral | 40 | 4.6000 | 13.60581 |
|  | Nosewitness | 40 | 39.0000 | 29.55308 |
| Happiness | Neutral | 40 | 31.0250 | 28.94778 |
|  | Nosewitness | 40 | 8.6000 | 16.58513 |
| Sadness | Neutral | 40 | 8.6500 | 17.86782 |
|  | Nosewitness | 40 | 39.8750 | 30.38487 |
| Sympathy | Neutral | 40 | 18.6000 | 24.67928 |
|  | Nosewitness | 40 | 44.7750 | 36.39209 |
| Disgust | Neutral | 40 | 7.0750 | 18.16221 |
|  | Nosewitness | 40 | 32.6750 | 28.95300 |
| Upset | Neutral | 40 | 5.1250 | 15.38929 |
|  | Nosewitness | 40 | 36.0750 | 28.75216 |
| Fright | Neutral | 40 | 2.6000 | 6.58981 |
|  | Nosewitness | 40 | 33.7250 | 31.64465 |
| Anxiety | Neutral | 40 | 10.6250 | 17.98673 |
|  | Nosewitness | 40 | 32.2750 | 32.54188 |
| Relief | Neutral | 40 | 17.4750 | 26.86432 |
|  | Nosewitness | 40 | 16.9250 | 24.14315 |

**Sex differences**

Chi-square tests did not show statistically significant differences between women and men for the witness and neutral conditions (*ps* > .05). In the nosewitness condition men performed 12 correct identifications (60%), compared to women who performed 8 correct identifications (40%). Moreover, in the neutral condition women had more correct identifications (7, corresponding to 33%) than men (4, 21%).

**Future studies**

The current study is the first to characterize nosewitness memory. There is much work to be done in order complete the picture. The observations that odor memory is enhanced for odors encoded during negative, compared to neutral, emotion merits further scrutiny.

Future studies should investigate effects of inter-individual differences in olfactory sensitivity and memory performance on nosewitness performance (e.g., [5]). Admittedly, our experiments drew on a mostly young population of students. A sample based on the general population may lead to somewhat different results.

Many aspects of the witnesses’ psychological or physiological state are pertinent to their memory performance. One example here is the level of stress the witness experiences while encoding of the body odor. Although higher emotional stress seems to boost memory performance in these studies, we do not know the effects of the extreme emotional stress that a real victim is likely to experience during a crime such as rape [6]. Moreover, recent studies have shown that the emotion of the BO *donor* could affect the perceiver through emotional contagion (e.g., [7]). It is likely that a perpetrator experiences emotional stress. Moreover, the menstrual cycle has been shown to alter a women’s sensitivity to smell and may therefore also prove to be of importance [8]. It is well worth investigating how these processes can affect the probability of recognizing a culprit.

When witnessing a crime involving an odor experience, one may or may not be aware of the odor. Odor awareness is likely to have effects on how the body odors are explicitly remembered [9,10] or otherwise to implicitly affect processing during exposure to the target body odor [11].

**References**

1. Kanazawa S (2009) Evolutionary psychology and crime. In: Walsh A, Kevin MB, editors. Biosocial criminology: New directions in theory and research. New York, NY: Routledge/Taylor and Francis Group. pp. 90–110.

2. Mitro S, Gordon AR, Olsson MJ, Lundström JN (2012) The smell of age: perception and discrimination of body odors of different ages. PLoS One 7: e38110. Available: http://www.pubmedcentral.nih.gov/articlerender.fcgi?artid=3364187&tool=pmcentrez&rendertype=abstract. Accessed 6 April 2014.

3. Spielberger CD (1983) Manual for the State-Trait Anxiety Inventory STAI (Form Y). Palo Alto: Consulting Psychologists Press.

4. Houston K a, Clifford BR, Phillips LH, Memon A (2013) The emotional eyewitness: the effects of emotion on specific aspects of eyewitness recall and recognition performance. Emotion 13: 118–128. Available: http://www.ncbi.nlm.nih.gov/pubmed/22775133. Accessed 26 May 2014.

5. Lundstrom JN, Hummel H, Olsson MJ (2003) Individual Differences in Sensitivity to the Odor of 4,16-Androstadien-3-one. Chem Senses 28: 643–650. Available: http://www.chemse.oupjournals.org/cgi/doi/10.1093/chemse/bjg057. Accessed 19 September 2014.

6. Deffenbacher K a., Bornstein BH, Penrod SD, McGorty EK (2004) A Meta-Analytic Review of the Effects of High Stress on Eyewitness Memory. Law Hum Behav 28: 687–706. Available: http://doi.apa.org/getdoi.cfm?doi=10.1007/s10979-004-0565-x. Accessed 26 March 2014.

7. De Groot JHB, Semin GR, Smeets M a M (2014) I can see, hear, and smell your fear: comparing olfactory and audiovisual media in fear communication. J Exp Psychol Gen 143: 825–834. Available: http://www.ncbi.nlm.nih.gov/pubmed/23855495. Accessed 13 October 2014.

8. Lundström JN, McClintock MK, Olsson MJ (2006) Effects of reproductive state on olfactory sensitivity suggest odor specificity. Biol Psychol 71: 244–247. Available: http://www.ncbi.nlm.nih.gov/pubmed/16084006. Accessed 4 September 2014.

9. Arshamian A, Willander J, Larsson M (2011) Olfactory awareness is positively associated to odour memory. J Cogn Psychol 23: 220–226. Available: http://www.tandfonline.com/doi/abs/10.1080/20445911.2011.483226. Accessed 3 September 2014.

10. Demattè ML, Endrizzi I, Biasioli F, Corollaro ML, Zampini M, et al. (2011) Individual Variability in the Awareness of Odors: Demographic Parameters and Odor Identification Ability. Chemosens Percept 4: 175–185. Available: http://link.springer.com/10.1007/s12078-011-9103-7. Accessed 19 September 2014.

11. Degel J, Köster EP (1999) Odors: implicit memory and performance effects. Chem Senses 24: 317–325. Available: http://www.ncbi.nlm.nih.gov/pubmed/10400450.

**Table legend**

**Table S1.** Means of the ratings for each emotion in the neutral and nosewitness conditions.
